# Custom-Made Ce–Mn Bimetallic Nanozyme for the Treatment of Intervertebral Disc Degeneration by Inhibiting Oxidative Stress and Modulating Macrophage M1/M2 Polarization

**DOI:** 10.34133/bmr.0118

**Published:** 2024-12-23

**Authors:** Jianwei Wu, Zhenhao Chen, Han Huang, Hongwei Wang, Xianghe Wang, Zian Lu, Haocheng Xu, Xiaosheng Ma, Feng Zeng, Hongli Wang

**Affiliations:** ^1^ Department of Orthopedics, Huashan Hospital, Fudan University, Shanghai 200000, China.; ^2^ Artemisinin Research Center, Guangzhou University of Chinese Medicine, Guangzhou 510450, China.

## Abstract

Intervertebral disc degeneration (IDD)-induced lower back pain (LBP) brings heavy burden worldwide. In the degenerated intervertebral disc, there is an increase in the accumulation of reactive oxygen species (ROS) and the infiltration of M1 macrophages, which leads to abnormal local inflammatory microenvironment and exacerbates IDD. In this study, we developed a novel injectable polyethylene glycol (PEG)-capped cerium ion–manganese ion (Ce–Mn) bimetallic nanozyme (CeMn-PEG) with strong ROS scavenging and M2-type macrophage polarizing abilities to efficiently alleviate IDD. In vitro experiments demonstrated that CeMn-PEG effectively scavenged excess ROS in both nucleus pulposus (NP) and RAW264.7 cells. In addition, we found that CeMn-PEG markedly protected NP cells from H_2_O_2_-induced overproduction of inflammatory cytokines, excessive cell apoptosis and autophagy, and imbalance between extracellular matrix (ECM) degradation. Moreover, CeMn-PEG induced macrophages to transition from the M1 phenotype to the M2 phenotype and the increased M2-type macrophages could alleviate H_2_O_2_-induced ECM degradation and cell apoptosis in NP cells. In a puncture-induced mouse IDD model, CeMn-PEG treatment could effectively ameliorate the progression of disc degeneration and mitigate puncture-induced mechanical hyperalgesia. Thus, our study demonstrated the effectiveness of CeMn-PEG as a novel treatment strategy for the treatment of IDD and a range of other inflammatory diseases.

## Introduction

Intervertebral disc degeneration (IDD), one of the major causes of lower back pain (LBP), leads to an enormous burden on both individuals and society [1,2]. Consequently, substantial efforts and resources have been spent on the prevention and treatment of IDD-related diseases. In clinical practice, conservative treatments for IDD-related diseases normally include physical therapy and pharmacological analgesia, while they can only relieve the symptoms and are not suitable for severe patients. Currently, surgical intervention is the only effective way to cure severe patients, while it can cause several severe complications including the increasing stress on adjacent disks and destruction of biomechanical function of spine [3]. Thus, there clearly is an urgent need to find more effective interventions for consistent and thorough solutions.

Intervertebral disc (IVD) consists of 3 parts: NP, annulus fibrosus (AF), and cartilage endplate (CEP) [4]. In degenerated IVD, the balance between extracellular matrix (ECM) anabolism and catabolism in the NP region is broken with the decrease of collagen II and aggrecan and up-regulation of a disintegrin and metalloproteinase with thrombospondin motif 5 (ADAMTS5) and matrix metalloproteinase 13 (MMP13) [5]. Although the pathogenesis of IDD is complicated and not fully understood, recent studies suggest that abnormal microenvironment of NP cells, especially the overactive inflammatory response and overproduced inflammatory cytokines [such as interleukin-1β (IL-1β), IL-6, and tumor necrosis factor-α (TNF-α)], disturbs the homeostasis of ECM and aggravates degenerative changes in IVD [6–8].

It has been reported that the overproduction of reactive oxygen species (ROS) in degenerated IVD contributes to the inflammatory microenvironment of NP cells via a series of redox signaling pathways, such as nuclear factor κB (NF-κB) and mitogen-activated protein kinase (MAPK) signaling pathways [9–11], and induces excessive NP cell apoptosis [12]. In addition, during the process of IDD, a number of immune cells, especially macrophages, infiltrates into the NP region [13,14]. Among the macrophages in degenerated IVD, pro-inflammatory M1-type macrophages constitute the vast majority, which exacerbate disc inflammation and degeneration [15,16]. Moreover, in the inflammatory microenvironment, elevated ROS level could induce the transformation of M1-type macrophages [17,18]. At the same time, the polarized M1-type macrophages subsequently induce more production of ROS, which leads to a vicious circle that exacerbates disc degeneration [13]. Thus, modulating local abnormal inflammatory microenvironment by eliminating ROS and polarizing anti-inflammatory M2-type macrophages is regarded as an effective way to maintain the homeostasis of ECM and alleviate the progression of IDD [19,20].

Researchers are currently developing a variety of nanomaterials as potent ROS scavengers for the treatment of various diseases, such as cancer [21], osteoarthritis [22], and rheumatoid arthritis [23], due to their excellent tunable composition, catalytic activity, physical stability, and special energy conversion ability compared with natural enzymes. Among these nanomaterials, metal element-based (Fe [24], Mn [25], Ce [26,27], and so on) nanozymes are widely developed as excellent antioxidant agents. Cerium is a rare earth metal, and cerium dioxide nanoparticles (CeNPs) have been investigated as antioxidant nanozymes for a variety of inflammatory diseases relying on the mixed valance states of 2 reversible oxidation states (Ce^3+^/Ce^4+^) on the surface of nanoparticles [26,27]. In our previous research, we found that custom-made CeNPs can protect neuronal cells from excessive ROS by polarizing microglial BV-2 cells [28]. In view of ROS scavenging activity and M2-like macrophage polarizing ability, CeNPs might be a great potential therapeutic application for ameliorating IDD. However, the ROS scavenging capability of most nanozymes including CeNPs is relatively moderate and high concentration of the CeNPs will lead to cytotoxicity [29,30]. Thus, how to develop more effective nano-antioxidants and reduce the necessary NP dose is of utmost importance and should be promptly addressed.

One effective strategy to improve ROS removal capability of CeNPs is adding other metal elements into nanoceria since the different radius between the doping metal element and cerium element can result in lattice distortion, variation of the lattice constants and more concentrated oxygen vacancy [31–33]. Numerous studies have reported that doping with manganese mental can effectively enhance the catalytic activity of nanoceria, and the manganese-doped ceria nanoparticles have been applied for the treatment of various diseases, such as Alzheimer’s disease [34] and intestinal ischemia–reperfusion injury [35]. However, to our knowledge, there are no reports concerning the therapeutic effect of manganese-doped ceria nanoparticles on IDD.

**Fig. 1.**
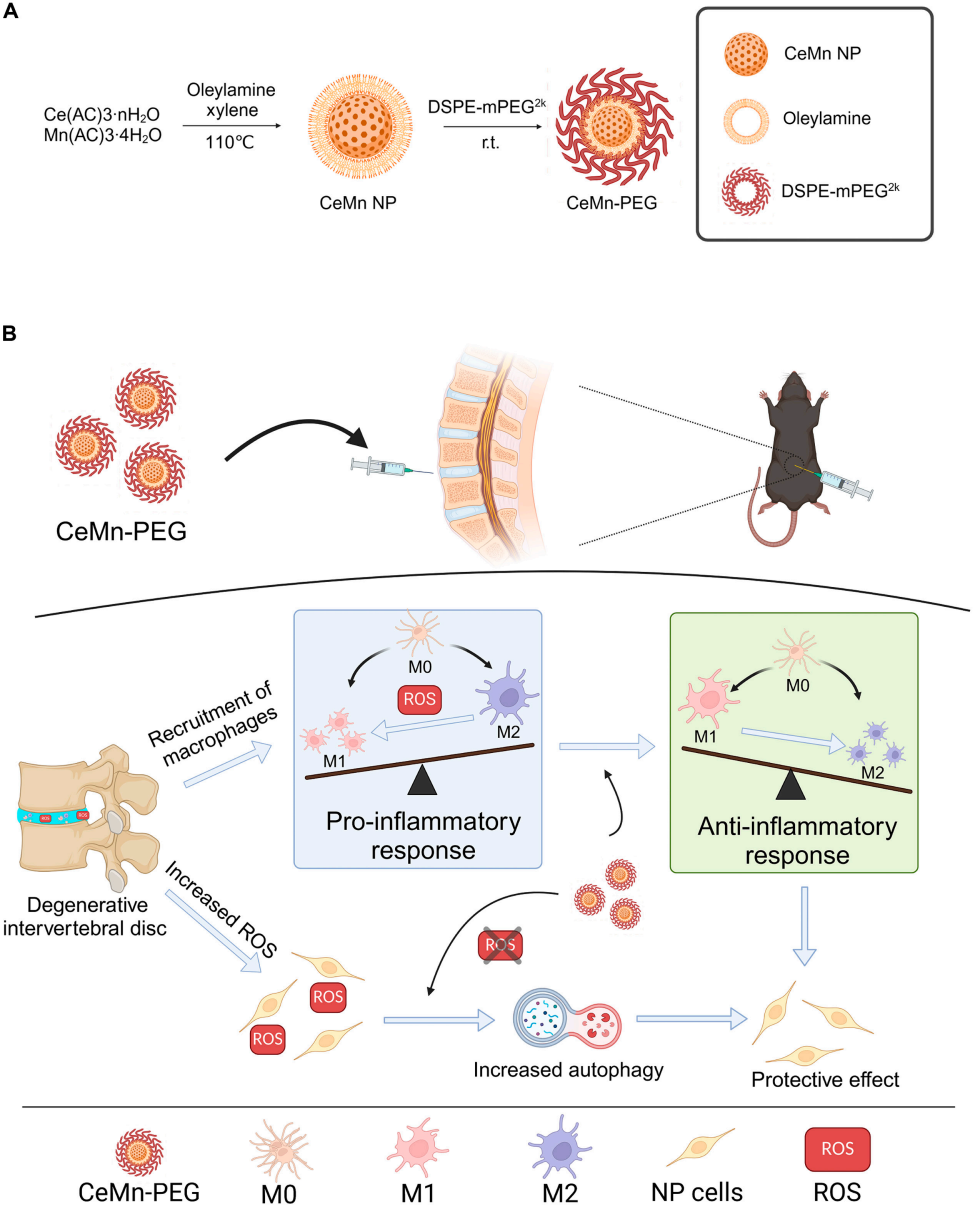
(A) Diagram of synthesis of CeMn NP and CeMn-PEG. (B) Schematic diagram of CeMn-PEG for the treatment of IDD in mouse.

Herein, we rationally synthesized polyethylene glycol (PEG)-capped Ce–Mn nanoparticles (CeMn-PEG) and first applied it for the treatment of IDD (Fig. 1). At the cellular level, we evaluated the ROS scavenging activity of CeMn-PEG on both NP and RAW264.7 cells after hydrogen peroxide (H_2_O_2_) treatment. Then, on the one hand, we assessed the protective effect of CeMn-PEG on H_2_O_2_-treated NP cells and investigated the underlying mechanism of this effect. On the other hand, we hypothesized that CeMn-PEG induced the polarization of M2-like macrophages, which further exerted protective effect on NP cells by regulating inflammatory response and NP cell apoptosis. Furthermore, in in vivo study, a classical model of the puncture-induced lumbar disk degeneration mouse model was established for determining the protective effect of CeMn-PEG on IDD.

## Results

### Formulation and characterization of CeMn NP and CeMn-PEG

**Fig. 2.**
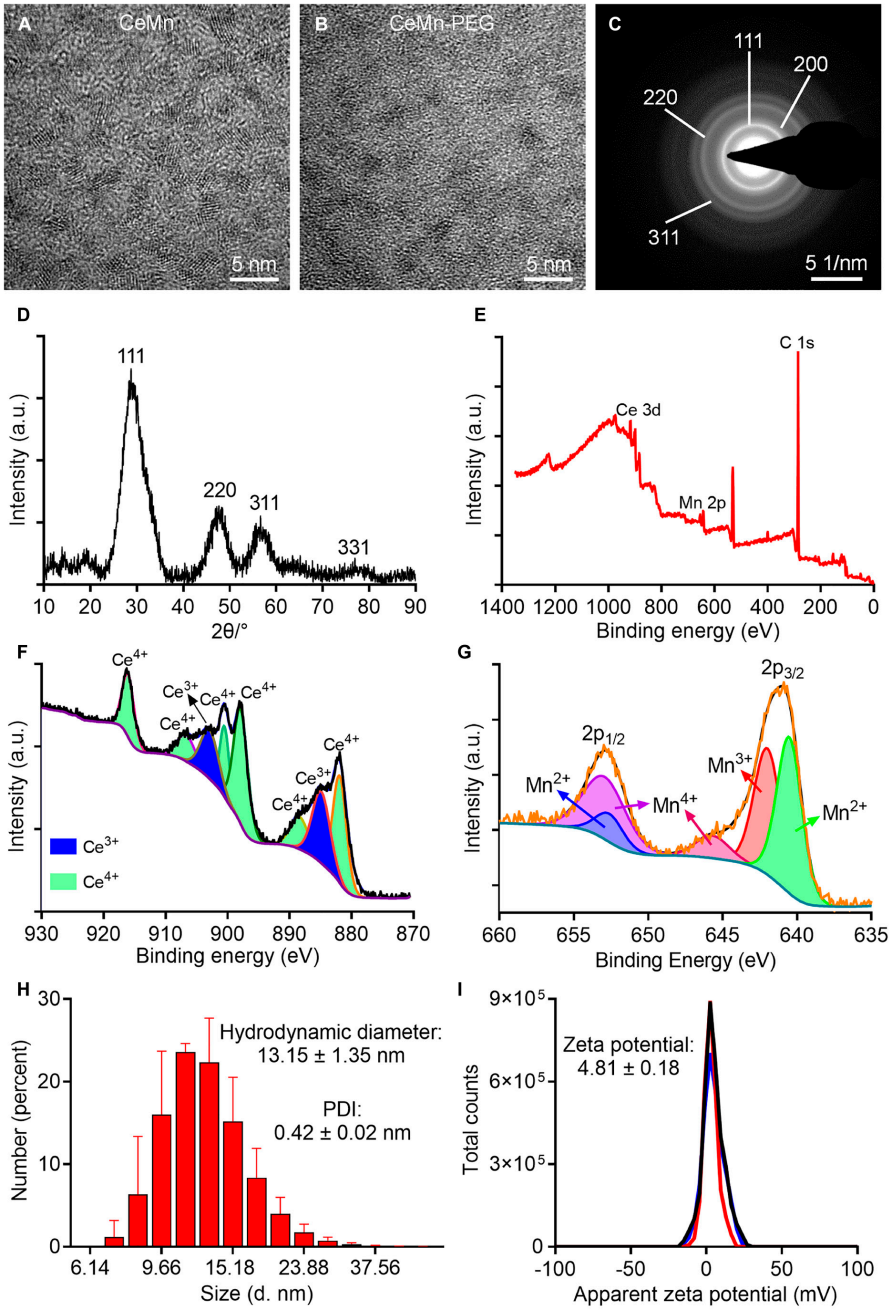
Characterization of CeMn NP and CeMn-PEG. Representative TEM image of CeMn NP (A) and CeMn-PEG (B). SAED pattern (C) and XRD (D) of CeMn. (E) Survey scan XPS spectrum of CeMn-NP. (F) XPS spectrum of Ce3d revealed the mixed valence states of Ce^3+^ (884.88 and 902.89 eV) and Ce^4+^ (881.89, 888.38, 897,88, 900.48, 906.66, and 916.03 eV). (G) XPS spectrum of Mn2p revealed the mixed valence states of Mn^2+^ (640.51 and 652.6 eV), Mn^3+^ (641.99 eV), and Mn^4+^ (645.69 and 652.91 eV). Hydrodynamic diameter (H) and zeta potential (I) of CeMn-PEG.

The bimetallic nanozyme CeMn nanoparticle (CeMn NP) and CeMn-PEG were synthesized by mixing Ce(AC)_3_, Mn(AC)_3_, and oleylamine xylene at 110 °C as shown in Fig. 1A. To improve the biocompatibility of CeMn NP for better biomedical applications, CeMn NPs were coated with 1,2-distearoyl-sn-glycero-3-phosphorylethanolamine (DSPE)-mPEG^2k^ to synthesize CeMn-PEG for better solubility. The representative transmission electron microscopy (TEM) images showed that CeMn NP had a uniform, discrete, and spherical morphology with a size of 1 to 2 nm a well as highly crystalline and cross-lattice patterns (Fig. 2A). As shown in Fig. 2B, the overall morphology and size of the CeMn-PEG NPs showed no obvious difference compared with that of the CeMn NP. The result of selected-area electron diffraction (SAED) patterns and x-ray diffraction (XRD) analyses also demonstrated that CeMn NP possessed highly crystalline structure and the well-indexed diffraction peaks of (111), (200), (220), and (311) (Fig. 2C and D). Image of Fig. 2E presented the x-ray photoelectron spectroscopy (XPS) spectrum of CeMn NP. Specifically, the XPS spectrum of Ce3d showed the mixed valence states of Ce^3+^ (peaks at 884.88 and 902.89 eV) and Ce^4+^ (peaks at 881.89, 888.38, 897,88, 900.48, 906.66, and 916.03 eV) (Fig. 2F), while the XPS spectrum of Mn2p revealed the mixed valence states of Mn^2+^ (peaks at 640.51 and 652.6 eV), Mn^3+^ (peaks at 641.99 eV), and Mn^4+^ (peaks at 645.69 and 652.91 eV) (Fig. 2G). The colloidal stability of CeMn-PEG was assessed via dynamic light scattering (DLS) by evaluating the hydrodynamic diameter and ζ-potential. As shown in Fig. 2H, the hydrodynamic diameter was 13.15 ± 1.35 nm with a polymer dispersity index (PDI) of 0.42 ± 0.02 nm, which was larger than the TEM size because of the coated PEG molecules on the appearance of CeMn NP. The ζ-potential value of CeMn-PEG was 4.81 ± 0.18 mV (Fig. 2I). The result of energy-dispersive spectrometry (EDS) analysis demonstrated that CeMn-PEG was composed of Ce, Mn, and O (Fig. S1).

ROS is a family of short-lived, evidently reactive, oxygen-containing molecules that mainly contain superoxide anion (O_2_**·**^−^), hydroxyl radical (**·**OH) and H_2_O_2_ [36]. Hydroxyl radical, superoxide anion scavenging ability, and CAT enzyme-like activity of CeNP-PEG were evaluated to assess the anti-ROS effect of CeMn-PEG. In addition, the antioxidant activity of CeNP-PEG was determined by ABTS [2,2′-azino-bis (3-ethylbenzothiazoline-6-sulfonic acid)] and DPPH (1,1-diphenyl-2-picrylhydrazyl) radical scavenging assays. As shown in Fig. S2A to D, CeMn-PEG showed excellent DPPH-free radical, ABTS radical, hydroxyl radical, and O_2_**·**^−^ scavenging ability in a dose-dependent manner. Meanwhile, the oxygen generation of H_2_O_2_ catalyzed by CeMn-PEG (6 and 12 μg/ml) was markedly higher than that without treatment of CeMn-PEG at different times, which was also related to the concentration of CeMn-PEG (Fig. S2E). In conclusion, these results indicated an excellent enzyme-like activity and antioxidant capacity of CeMn-PEG.

### Cytocompatibility and ROS scavenging activity of CeMn-PEG on RAW264.7 and NP cells

**Fig. 3.**
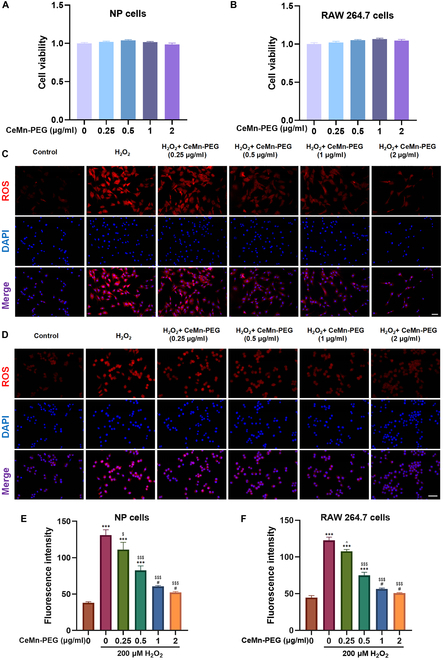
The biocompatibility and ROS scavenging properties of CeMn-PEG in vitro. (A and B) Effects of various concentrations of CeMn-PEG (0, 0.25, 0.5, 1, and 2 μg/ml) on the viability of both NP and RAW264.7 cells after incubation for 24 h by using CCK8 assay. (C to F) Representative ROS fluorescence microscopy images and relative fluorescence intensity of NP cells (C and E) and RAW264.7 cells (D and F) pretreated with various concentrations of CeMn-PEG (0, 0.25, 0.5, 1, and 2 μg/ml) after the treatment of H_2_O_2_ (200 μM) for 6 h. Scale bar, 25 μm. Data are presented as the mean ± SEM. ^#^*P* > 0.05, *P < 0.05, **P < 0.01, ***P < 0.001 relative to the control group; ^*P* > 0.05, ^$^*P* < 0.05, ^$$^*P* < 0.01, ^$$$^*P* < 0.001 relative to the H_2_O_2_-treated group, *n* = 3.

An excellent biocompatibility is essential for the application of the nanozyme. The cytotoxic effects of CeMn-PEG on NP and RAW264.7 cells were evaluated using the CCK8 (Cell Counting Kit 8) assay, with various concentrations (0, 0.25, 0.5, 1, and 2 μg/ml) administered for 24 h. As shown in Fig. 3A and B, CeMn-PEG was not toxic to both NP and RAW264.7 cells at all concentrations (0, 0.25, 0.5, 1, and 2 μg/ml).

Oxidative stress is generated when the redox homeostasis between ROS generation and ROS scavenging is disturbed, causing a series of cellular dysfunctions and leading to various diseases [37–39]. As previously reported, elevated levels of ROS notably contribute to the progression of IDD [40–42]. Therefore, we employed 200 μM H_2_O_2_ to simulate the oxidative stress microenvironment in vitro and assessed the ROS scavenging capacity of CeMn-PEG in NP and RAW264.7 cells by using fluorescing dihydroethidium (DHE) assay. As expected (Fig. 3C to F), H_2_O_2_ stimulation substantially up-regulated ROS levels in NP and RAW264.7 cells, while excessive ROS could be obviously inhibited when pretreated with different concentrations (0.25, 0.5, 1, and 2 μg/ml) of CeMn-PEG. Considering that ROS fluorescence intensity of the 1 and 2 μg/ml CeMn-PEG pretreated groups showed no significant difference compared with that of the control group, 1 μg/ml CeMn-PEG was used in subsequent experiments. All in all, our result demonstrated that CeMn-PEG had good biocompatibility and strong ROS scavenging activity on RAW264.7 and NP cells.

### CeMn-PEGs ameliorate H_2_O_2_-induced inflammation, ECM degradation, and cell apoptosis in vitro

**Fig. 4.**
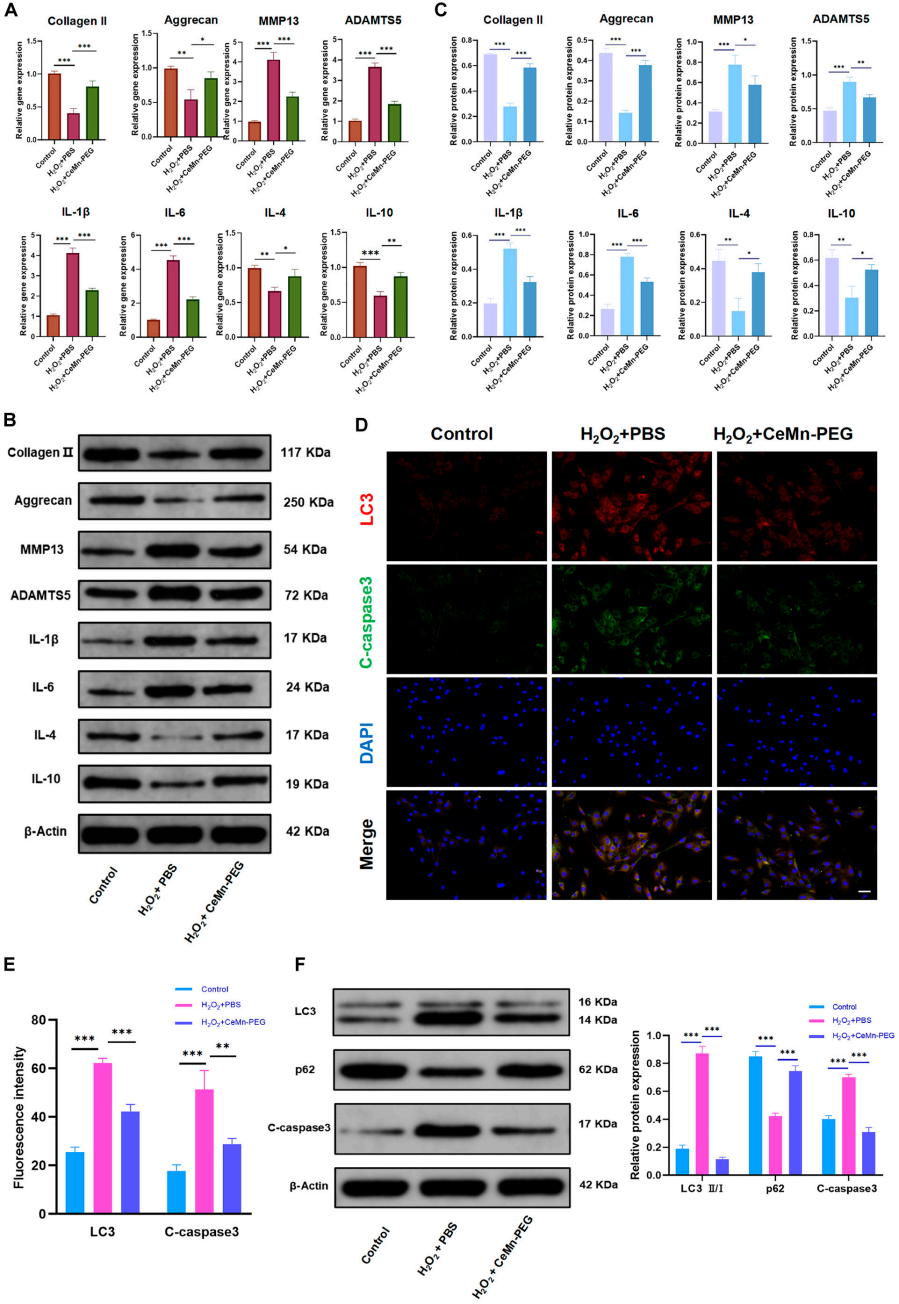
CeMn-PEG ameliorated H_2_O_2_-induced inflammation and ECM degradation and reduced H_2_O_2_-induced apoptosis and autophagy in vitro. (A) The relative mRNA expression of IL-1β, IL-4, IL-6, IL-10, collagen II, aggrecan, MMP13, and ADAMTS5 was determined by using qPCR. (B and C) Representative images and quantification data of Western blot results of IL-1β, IL-4, IL-6, IL-10, collagen II, aggrecan, MMP13, and ADAMTS5. (D and E) Representative images and relative fluorescence intensity of double immunofluorescence of LC3 (red) and C-caspase3 (green) in NP cells. (F) Representative images and quantification data of Western blot results of LC3, p62, and C-caspase3. Scale bar, 25 μm. Data are presented as the mean ± SEM. *P < 0.05, **P < 0.01, ***P < 0.001, *n* = 3.

Numerous studies have reported that excessive ROS resulted in the imbalance between ECM catabolism and anabolism with decreased anabolic related proteins (collagen II and aggrecan) and increased degrading enzymes (MMP13 and ADAMTS5) in NP cells [43,44]. As expected (Fig. 4A to C), the Western blot results and quantitative reverse transcription polymerase chain reaction (qRT-PCR) results showed that the gene expressions and protein levels of collagen II and aggrecan were substantially decreased after H_2_O_2_ stimulation, while the gene expressions and protein levels of MMP13 and ADAMTS5 were increased. However, our results showed that pretreatment with CeMn-PEG reversed these alterations induced by H_2_O_2_ and retained the ability to synthesize the ECM (Fig. 4A to C). Considering that overproduced pro-inflammatory chemokines played pivotal roles during the pathological process of IDD, we next investigated the gene expressions and protein levels of IL-1β, IL-4, IL-6, and IL-10 in NP cells. The Western blot results and qRT-PCR results showed that pro-inflammatory chemokine (IL-1β and IL-6) levels increased, and anti-inflammatory chemokine (IL-4 and IL-10) levels declined after H_2_O_2_ stimulation. However, CeMn-PEG pretreatment markedly reversed these alternations.

ROS, a crucial proapoptotic factor, has been reported to induce excessive cell apoptosis and therefore contributes to the development of IDD [45,46]. As expected, after incubation with H_2_O_2_, the apoptosis rate (Fig. S3A and B) and expression of apoptosis-related protein [cleaved caspase3 (C-caspase3)] (Fig. 4D to F) in NP cells were markedly increased. When NP cells were pretreated with CeMn-PEG, the apoptosis rate decreased from 26.04% to 14.19% (Fig. S3A and B). Simultaneously, Western blot analysis and fluorescence staining demonstrated that CeMn-PEG pretreatment could reverse the increased expression of C-caspase3 induced by H_2_O_2_ stimulation (Fig. 4D to F). In addition, it has been widely reported that loss of mitochondrial membrane potential (MMP) is a hallmark event in the early stages of apoptosis. Thus, the MMP in NP cells was detected for evaluating cell apoptosis by flow cytometry using JC-1 staining. The results of flow cytometric analysis of JC-1 staining exhibited that H_2_O_2_ treatment led to the decrease of MMP, while CeMn-PEG pretreatment increased the level of MMP in H_2_O_2_-treated NP cells (Fig. S3C and D). Collectively, these results showed that CeMn-PEG alleviated H_2_O_2_-induced inflammation, ECM degradation, and cell apoptosis in NP cells.

### CeMn-PEG reduced H_2_O_2_-induced apoptosis by down-regulating autophagy

Several studies have indicated that ROS is crucial in regulating autophagy in disc cells [47–49], and Chen et al. [12] demonstrated that prototypic ROS (H_2_O_2_) resulted in apoptosis by inducing autophagy in NP cells. LC3 and p62 are regarded as key markers of autophagy formation. The data presented in Fig. 4D to F indicated that, relative to the control group, Western blot analysis and fluorescence staining exhibited an increase in LC3II/I levels and a decrease in p62 expression in NP cells treated with H_2_O_2_. This suggested that oxidative stress promoted autophagy in NP cells. However, CeMn-PEG pretreatment substantially reduced autophagy level (decreased LC3II/I expression and increased p62 expression) induced by H_2_O_2_ stimulation. Autophagy inducer, rapamycin, was used to evaluate whether CeMn-PEG reduced H_2_O_2_-induced apoptosis of NP cells by inhibiting autophagy. The administration of rapamycin elevated the apoptosis rate and MMP level of NP cells with the treatment of H_2_O_2_ and CeMn-PEG (Fig. S3A to D), indicating that inducing autophagy could reverse the inhibitory effect of CeMn-PEG on H_2_O_2_-induced apoptosis of NP cells. Taken together, these results demonstrated that pretreatment with CeMn-PEG could reduce H_2_O_2_-induced apoptosis by inhibiting autophagy.

### RAW264.7 cells pretreated with CeMn-PEG could alleviate H_2_O_2_-induced ECM degradation and apoptosis in NP cells

**Fig. 5.**
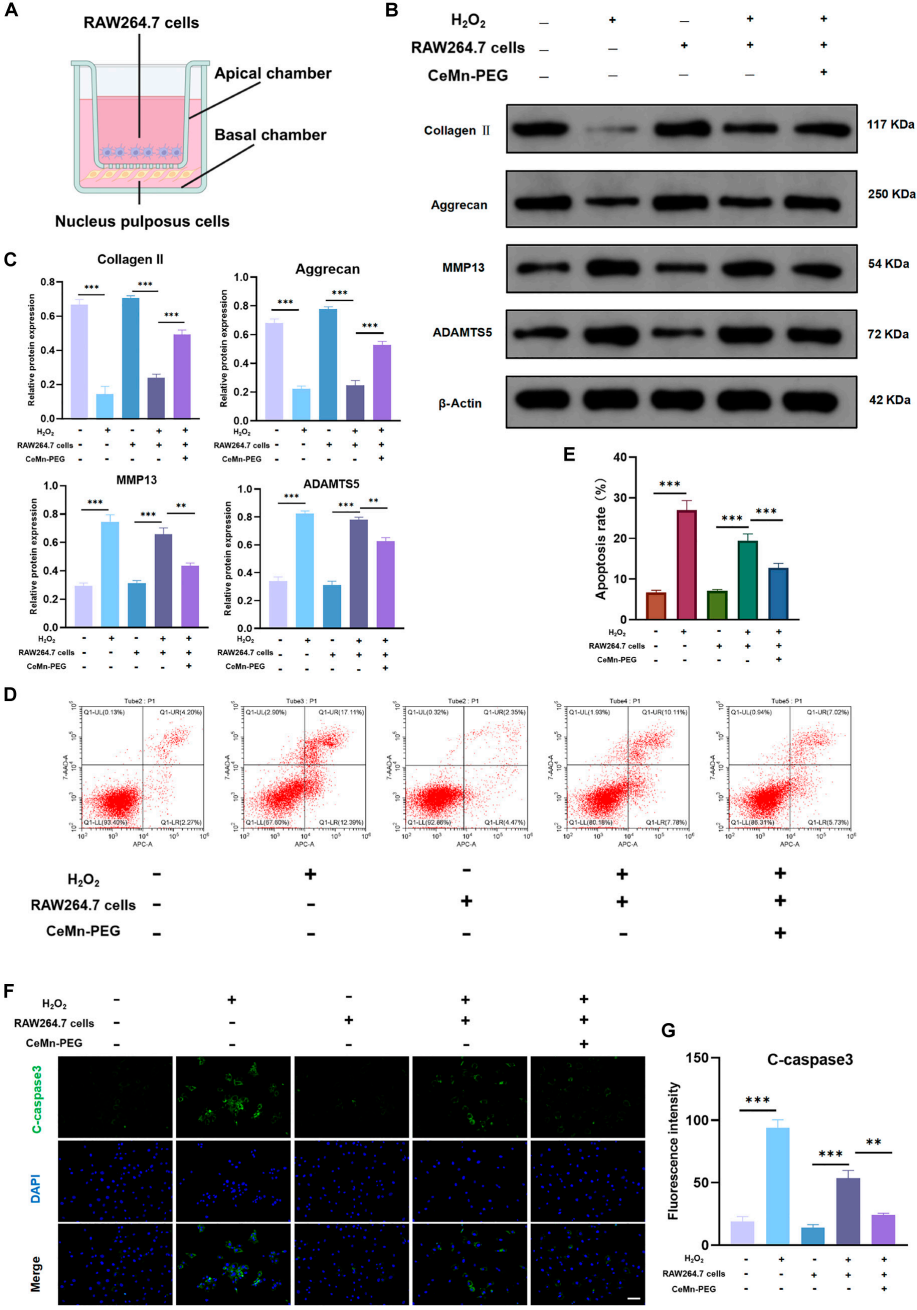
RAW264.7 cells pretreated with CeMn-PEG could alleviate H_2_O_2_-induced ECM degradation and apoptosis in NP cells. (A) Diagram of RAW264.7 cells (apical chamber) and NP cells (basal chamber) cocultured by using Transwell dish. RAW264.7 cells were pretreated with CeMn-PEG and then cocultured with NP cells. (B and C) Representative images and quantification data of Western blot results of collagen II, aggrecan, MMP13, and ADAMTS5 in NP cells in the coculture system. (D) Representative images of flow cytometry analysis of NP cells in the coculture system. (E) Apoptosis rate of NP cells in the coculture system estimated by flow cytometry analysis. (F and G) Representative images and relative fluorescence intensity of C-caspase3 (green) in NP cells in the coculture system. Scale bar, 25 μm. Data are presented as the mean ± SEM. *P < 0.05, **P < 0.01, ***P < 0.001, *n* = 3.

It has been reported that infiltration of macrophages in NP contributes to NP degeneration and painful inflammatory response [50–52]. Therefore, we aimed to evaluate the influence of CeMn-PEG on macrophages as well as the further effect of macrophages on NP cells. To investigate the effect of RAW264.7 cells pretreated with CeMn-PEG on NP cells under oxidative stress in vitro, NP cells were cocultured with RAW264.7 cells in transwell dish (Fig. 5A). Our results showed that RAW264.7 cells pretreated with CeMn-PEG substantially inhibited H_2_O_2_-induced apoptosis and ECM degradation of NP cells. The Western blot results showed that coculturing with RAW264.7 cells pretreated with CeMn-PEG elevated the expression of anabolic related proteins (collagen II and aggrecan), accompanied with the decreased degrading enzymes such as MMP13 and ADAMTS5 in NP cells (Fig. 5B and C). In addition, compared with the effect of RAW264.7 cells on NP cells, RAW264.7 cells pretreated with CeMn-PEG obviously decreased apoptosis and the expression of C-caspase3 of NP cells (Fig. 5D to G). These results demonstrated that coculturing with RAW264.7 cells pretreated with CeMn-PEG could effectively alleviate H_2_O_2_-induced ECM degradation and apoptosis in NP cells.

### CeMn-PEG could promote the polarization of macrophages to M2 type

**Fig. 6.**
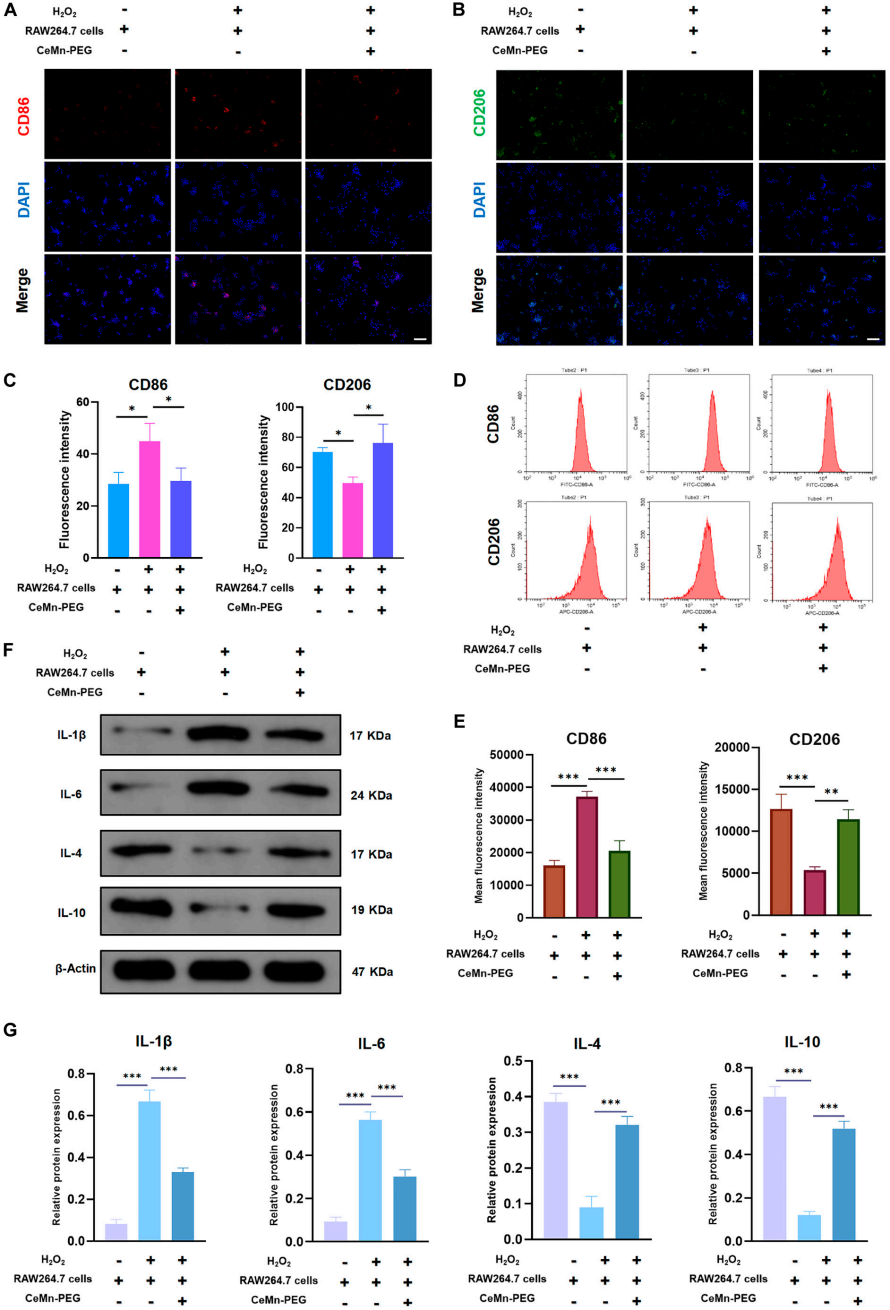
CeMn-PEG could promote the polarization of macrophages to M2 type. (A to C) Representative images and relative fluorescence intensity of CD86 and CD206 in RAW264.7 cells in the coculture system. (D and E) Representative images of cytometry analysis (D) and mean fluorescence intensity (MFI) quantification (E) of CD86 and CD206 in RAW264.7 cells in the coculture system. (F and G) Representative images and quantification data of Western blot results of IL-1β, IL-4, IL-6, and IL-10 in RAW264.7 cells in the coculture system. Scale bar, 25 μm. Data are presented as the mean ± SEM. *P < 0.05, **P < 0.01, ***P < 0.001, *n* = 3.

Considering the protective effect of RAW264.7 cells pretreated with CeMn-PEG on NP cells, we suspect that CeMn-PEG exerts protective effect by modulating the M2 macrophage polarization. As expected, the result of immunofluorescence assay (Fig. 6A to C) and flow cytometry (Fig. 6D and E) showed that H_2_O_2_-treated macrophages exhibited typical M1 phenotype with up-regulated expression of CD86 and down-regulated expression of CD206, while pretreatment of CeMn-PEG inhibited “M1” polarization and promoted “M2” polarization of RAW264.7 cells, characterized by the decreased CD86 and increased CD206. In addition, qRT-PCR results showed that pretreatment of CeMn-PEG down-regulated pro-inflammatory M1 genes (IL-1β and IL-6) and up-regulated anti-inflammatory M2 genes (IL-4 and IL-10) in RAW264.7 cells after H_2_O_2_ stimulation (Fig. 6F and G), which was in line with the changes of phenotype markers. Overall, these results suggested that CeMn-PEG reversed the “M2” phenotype to “M1” phenotype polarization tendency induced by H_2_O_2_ stimulation in RAW264.7 cells and these “M2” phenotype polarization might contribute to the protective effect of RAW264.7 cells pretreated with CeMn-PEG on NP cells.

### CeMn-PEG could ameliorate the puncture-induced degeneration of mouse IVD in vivo

**Fig. 7.**
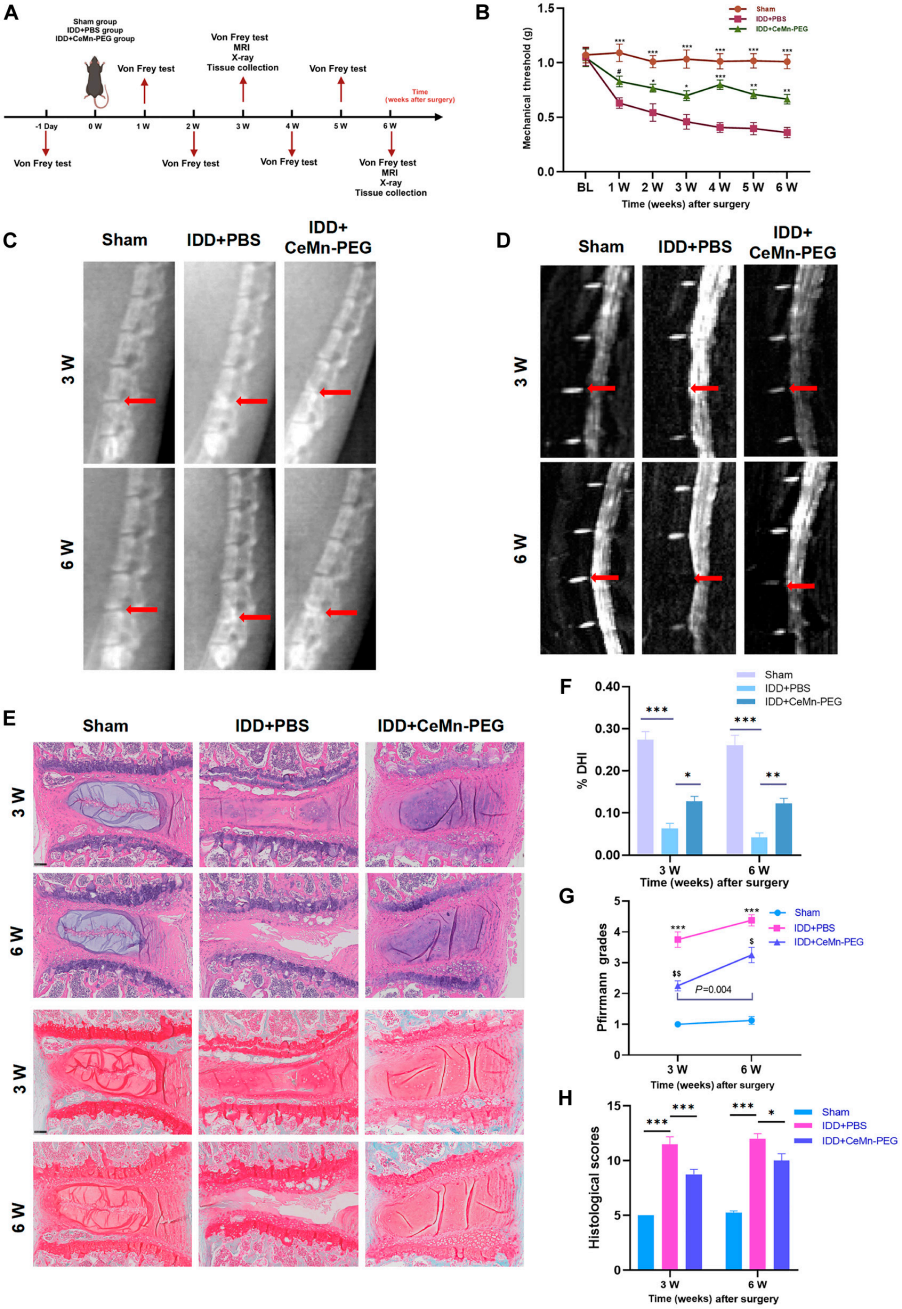
CeMn-PEG could ameliorate the puncture-induced degeneration of mouse IVD in vivo. (A) Schematic graph exhibits in vivo experimental procedure including the establishment of the puncture-induced mouse IVD model with injection of 5 μl of PBS or CeMn-PEG (1 μg/ml) and arrangement of behavior test (von Frey test) and imaging examination (MRI and x-ray). Mice were harvested at 3 and 6 weeks after surgery. (B) Paw withdrawal threshold of mice by using von Frey test. Data are presented as the mean ± SEM. ^#^*P* > 0.05, *P < 0.05, **P < 0.01, ***P < 0.001 relative to the IDD + PBS group, *n* = 8. (C) Representative radiographs of needle-punctured mouse lumbar disc of each group at 3 and 6 weeks after surgery (red arrows). (D) Representative T2-weighted MRI images of needle-punctured mouse lumbar disc of each group at 3 and 6 weeks after surgery (red arrows). (E) Representative H&E staining and Safranin O/Fast Green staining of disc samples in each group at 3 and 6 weeks after the operation. Scale bar, 100 μm. (F) Disc height index (%DHI) of needle-punctured mouse lumbar disc evaluated according to radiographs in 3 groups at 3 and 6 weeks after surgery. Data are presented as the mean ± SEM. *P < 0.05, **P < 0.01, ***P < 0.001, *n* = 8. (G) Pfirrmann grade scores of needle-punctured mouse lumbar disc evaluated according to MRI images in 3 groups at 3 and 6 weeks after surgery. Data are presented as the mean ± SEM. ***P < 0.001 relative to the IDD + PBS group, *n* = 8. ^$^*P* < 0.05, ^$$^*P* < 0.01 relative to the sham group, *n* = 8. (H) Histological grades of needle-punctured mouse lumbar disc evaluated according to H&E staining and Safranin O/Fast Green staining in 3 groups at 3 and 6 weeks after surgery. Data are presented as the mean ± SEM. *P < 0.05, **P < 0.01, ***P < 0.001, *n* = 8.

We successfully established a mouse model of IDD by needling the vertebral disc of L5–L6, and then von Frey test, calcitonin gene related peptide (CGRP) immunofluorescence staining, magnetic resonance imaging (MRI), x-ray assessment, and histopathologic analysis were used to evaluate the therapeutic effect of CeMn-PEG after disc puncture and injection with phosphate-buffered saline (PBS) (5 μl) or CeMn-PEG (1 μg/ml, 5 μl) (Fig. 7A). Previous studies have demonstrated that annular puncture of lumbar IVD could induce a decrease of paw withdrawal threshold in mechanical hyperalgesia test and an increased CGRP-positive nociceptive DRG neurons, which were reported to be associated with puncture-induced LBP [53–55]. As in previous studies, the results of von Frey test showed that paw withdrawal threshold in the IDD + PBS group substantially decreased compared with that of the sham group after surgery, while treatment of CeMn-PEG attenuated the tactile allodynia induced by disk puncture (Fig. 7B). As expected, CGRP immunofluorescence staining in DRG sensory neurons also indicated an increased expression of CGRP in the IDD + PBS group relative to the sham group, but CeMn-PEG administration reduced its expression (Fig. S4). In addition, we observed the most severe disc degeneration in the IDD + PBS group via x-ray and MRI assessment and this change was partly reversed by the treatment of CeMn-PEG as reflected by the recovery of decreased disc height and reduced disc T2-weighted intensity (Fig. 7C, D, F, and G). Moreover, histologic results as shown in (Fig. 7E) displayed destruction of the disc structure and reduced disk height in the IDD + PBS group, while these degenerative changes were substantially reversed by the treatment with CeMn-PEG. Meanwhile, the histological scores of the CeMn-PEG group were obviously lower than those of the IDD + PBS group at 3 and 6 weeks after surgery (Fig. 7H). However, the results of the von Frey test, x-ray assessments, and histopathological analyses showed no significant differences in puncture-induced degeneration within the CeMn-PEG treatment group between 3 and 6 weeks after surgery. In contrast, MRI assessments indicated that the Pfirrmann grades at 6 weeks after surgery were markedly higher than those at 3 weeks, suggesting a progression to more severe disk degeneration in the CeMn-PEG treatment group. All these data revealed that CeMn-PEG could inhibit ECM degradation and ameliorate the puncture-induced degeneration of mouse IVD in vivo.

## Discussion

The IVD is a fibrocartilage structure with inner colloidal NP that maintains proper spinal function. Normally, healthy IVD is considered as an avascular and immune-privileged tissue without any immune cells. However, during the process of IDD, immune cells, especially macrophages, will infiltrate into the “non-intact” IVD through new blood vessels resulted by the rupture or damage of AF or CEP [51,52,56]. The accumulating macrophages in the IVD, especially the M1-phenotype macrophages, constantly activate chronic inflammatory responses and secrete pro-inflammatory cytokines, such as IL-1β and IL-6, which further exacerbate the disk degeneration and lead to LBP [16,57,58]. In addition, accumulating evidence have revealed that NP cells are exposed to ROS abundant environment in degenerative discs and the excessive ROS plays an important role in the pathogenesis of IDD by inducing inflammatory microenvironment, disc cell apoptosis and ECM degradation [45,46]. Furthermore, accumulated inflammatory cytokines and ROS in degenerative discs further recruited M1-phenotype macrophages, which may dramatically active the positive feedback loop of inflammation responses [19]. Thus, how to develop a therapeutic method that combines the ROS scavenging and M2-phenotype polarizing ability in degenerative discs for the treatment of IDD has emerged as a challenge for researchers.

Recently, several nanozymes with capability of ROS scavenging activity have displayed potential application in the treatment of IDD. Shi et al. [59] explored the greigite nanozyme and found that the greigite nanozyme could alleviate IDD by inhibiting senescence through the ROS–p53–p21 pathway. Wu et al. [60] developed a novel ROS scavenging nanozyme called N-acetylcysteine-derived carbon dots nanozyme (NAC-CDs), which exhibited strong anti-oxidative function in the IDD model. Although currently the Mn-based and Ce-base nanozymes with antioxidant activities have been applied in various diseases such as osteoarthritis [25], renal fibrosis [26], Alzheimer’s disease [61], and rheumatoid arthritis [62], these nanozymes have not been utilized for the treatment of IDD. Thus, our study intended to synthesize Ce–Mn nanozymes with enhanced multi-antioxidant and M2-phenotype polarizing ability for the treatment of IDD.

The diameter of CeMn NP was nearly in the range of 1 to 2 nm, which increased its pseudo-enzyme activity due to its tiny size and high specific surface area. After coating with DSPE-mPEG^2k^, CeMn-PEG showed an excellent colloidal stability and better solubility. In addition, we confirmed enzyme-like activity and antioxidant capacity of CeMn-PEG including robust DPPH-free radical, ABTS radical, hydroxyl radical, superoxide anion scavenging ability and CAT enzyme-like activity. The sustainable and enhanced capacity of CeMn-PEG to scavenge multiple ROS can be attributed to several key factors: (a) Dual mechanism of action: Ce^3+^ is effective in eliminating superoxide (O₂•^-^) and hydroxyl radicals (•OH) [63,64], while Ce^4+^ is responsible for degrading H₂O₂ [65]. The oxidation–reduction cycle between Ce^3+^ and Ce^4+^ within biological systems allows CeMn-PEG to simultaneously and sustainably eradicate various types of ROS. (b) Manganese doping: Numerous studies have demonstrated that doping with manganese can notably enhance the ROS activity of nanoceria [34,35,66]. This enhancement is due to the introduction of more vacancy defects and improved electron transfer rates. In summary, the sustainable and enhanced multiple ROS scavenging ability of CeMn-PEG arises from the synergistic combination of cerium (Ce) and manganese (Mn) elements.

After various enzyme-like activities and antioxidant capacity of CeMn-PEG were determined, the therapeutic effect of CeMn-PEG for the treatment of IDD was further evaluated in vivo and in vitro. Our results indicated that CeMn-PEG (0.25, 0.5, 1, and 2 μg/ml) showed no obvious cytotoxic effects on both NP and RAW264.7 cells, while they exhibited excellent ROS scavenging ability after H_2_O_2_ treatment. Considering that 1 μg/ml CeMn-PEG has reached ideal ROS eliminating effect, we used 1 μg/ml CeMn-PEG in the following experiments.

Consistent with previous studies, our results demonstrated that H_2_O_2_ treatment induced overproduction of inflammatory cytokines, imbalance of synthesis/catabolism of ECM, and excessive apoptosis, while pretreatment with CeMn-PEG could effectively reverse these alternations. In addition, excessive ROS generated in degenerative discs have been reported to enhance NP cell autophagy [12]. As expected, our results showed that H_2_O_2_ treatment up-regulated the level of autophagy in NP cells with increased expression of LC3II/I and decreased expression of p62. Our results further demonstrated that enhancing autophagy with the application of autophagy inducer (rapamycin) could increase apoptosis compared with the H_2_O_2_ + CeMn-PEG group. Thus, we concluded that CeMn-PEG could protect against apoptosis in NP cells via autophagy inhibition.

It is worth mentioning that the effect of H_2_O_2_-induced autophagy on NP cells still remains controversial. Chen et al. [12] reported that treatment of H_2_O_2_ reduced autophagy and induced cell senescence and apoptosis [67]. However, similar to Chen et al.’s study [12], our results showed that H_2_O_2_ treatment markedly induced autophagy and apoptosis, while these effects were partially attenuated with the treatment of CeMn-PEG. These different conclusions could be attributed to the degree of oxidative stress, which was determined by the concentration and administered time of H_2_O_2_.

In addition to the direct protection of CeMn-PEG on NP cells after H_2_O_2_ treatment by improving the oxidative environment, we reported the protective effect of RAW264.7 cells pretreated with CeMn-PEG on NP cells by using a cell cocultured system. Previous studies have demonstrated that in human and murine IVDs, infiltration of macrophages was discovered in degenerated discs and the number of macrophages was associated with the degenerated degree of discs [50,51,68]. Moreover, inducing macrophages to transition from the M1 phenotype into the M2 phenotype has been reported as an effective method to promote IVD tissue regeneration [19,58,69–71], which could reduce the production of pro-inflammatory factors (IL-1β and IL-6) and increase the production of anti-inflammatory factors (IL-4 and IL-10). Similarly, in our study, we demonstrated that CeMn-PEG modulated the M2 macrophage polarization, which further led to decreased expression of pro-inflammatory genes, increased expression of anti-inflammatory genes and reduced apoptosis of NP cells.

In in vivo study, the lumbar disk puncture-induced degeneration mouse model was established for evaluating the therapeutic effects of CeMn-PEG. At 3 and 6 weeks after puncturing, we observed lower T2-weighted signal intensities, reduced disk height, and more severe destruction of the disc structure in the punctured group compared with the sham group, while these alterations partially attenuated with the treatment of CeMn-PEG. In addition, the administration of CeMn-PEG obviously mitigated puncture-induced allodynia and decreased the expression of CGRP in DRG. These results indicated that CeMn-PEG may be a potential therapeutic method for the treatment of IDD. Considering that IVD was an avascular organ and orally administered CeMn-PEG might not be able to exert its effect for the treatment of IDD, local disc injection with CeMn-PEG was used in our study for effective delivery. Because CeMn-PEG cannot reach to other organ such as liver, lung, and renal due to the local injection, in this study we only evaluated the biocompatibility of CeMn-PEG in vitro.

It is worth mentioning that during the ROS scavenging process of CeMn-PEG, a certain amount of oxygen is produced. To address the influence of oxygen (O₂) production during the ROS scavenging process of CeMn-PEG, we will examine 2 scenarios: optimal O₂ production and overproduction of O₂ in degenerated IVDs. Zhang et al. [72] propose that IDD is characterized by a chronic process involving a progressive decline in nutrient supply to the IVD. This decline adversely affects the maintenance of the ECM, resulting in further reductions in oxygen concentration within the IVD. In this context, optimal O₂ production can provide an essential oxygen equivalent for NP cells, improving the low oxygen levels in the microenvironment and subsequently restoring tissue strength and cellular metabolism. However, excessive O₂ production may disrupt the hypoxic microenvironment critical for NP cell function. Exposure to elevated oxygen tension has been shown to effectively increase ROS production [73,74], thereby enhancing matrix catabolism and autophagy [75,76]. Notably, CeMn-PEG, along with other ROS-scavenging biomaterials exhibiting robust catalase (CAT) activity [77–80], has been reported as an effective approach to mitigate IDD despite the concomitant production of O₂. These biomaterials may help alleviate the negative effects associated with excessive O₂ on NP cells by ROS scavenging. Furthermore, our study specifically focuses on the protective effects of CeMn-PEG against IDD by inhibiting oxidative stress and modulating macrophage M1/M2 polarization. Thus, it is plausible that CeMn-PEG may utilize additional mechanisms to counteract the adverse effects of oxygen.

Our study also had some limitations. First, our result demonstrated that CeMn-PEG could promote the polarization of macrophages to M2 type, while the detailed mechanisms of how CeMn-PEG increased the ratio of M1/M2 macrophages still remain unclear. In addition, in in vivo study, our result indicated that CeMn-PEG obviously alleviated IDD and mitigated puncture-induced allodynia, ignoring exploring the specific mechanism of how CeMn-PEG exerted this effect. Previous studies have reported that lumbar disk puncture could induce LBP with behavioral signs such as decreased paw withdrawal threshold, accompanied with the increased number of CGRP-positive neuropeptides in DRGs [53–55]. Although the pathogenesis of LBP has not been fully elucidated, accumulated M1 pro-inflammatory macrophages in disk have been reported to be associated with LBP through initiation or perpetuation of inflammatory cascades [81]. Thus, the mechanism of how CeMn-PEG alleviates IDD and puncture-induced pain is a difficult problem that is yet to be adequately resolved. Additionally, the MRI results revealed a more severe disk degeneration in the CeMn-PEG treatment group at 6 weeks after surgery compared to 3 weeks after surgery. This observation may be explained by the local injection of CeMn-PEG, which does not appear to provide a long-term therapeutic effect on IDD, likely due to the degradation of CeMn-PEG by 6 weeks after surgery. Consequently, further research is needed to develop a sustained delivery system that can prolong the effective therapeutic duration of CeMn-PEG.

## Conclusion

In this study, we developed a novel bimetallic nanozyme CeMn-PEG that can regulate H_2_O_2_-induced local inflammatory microenvironment, cell apoptosis and ECM degradation by scavenging ROS and inducing macrophage M2 polarization with excellent biocompatibility. Furthermore, CeMn-PEG treatment effectively alleviated the IDD process in a puncture-induced mouse IDD model.

## Methods

### Synthesis of CeMn NP and CeMn-PEG

CeMn NPs and CeMn-PEG were synthesized following previously established protocols with several modifications [26]. First, cerium (II) acetate hydrate (190 mg, 0.6 mM) and manganese (II) acetate tetrahydrate (98 mg, 0.4 mM) were dissolved in a 30-ml mixture of oleylamine (15 ml) and xylenes (15 ml). The solution was sonicated and stirred at room temperature until it became clear and transparent, indicating complete dissolution of the metal complexes and achieving a homogeneous solution. After heating to 110 °C under N_2_ protection, 1 ml of ddH_2_O was injected into the solution, and the mixture was aged at 110 °C for 3 h in N_2_ atmosphere. After cooling down, the CeMn NP was precipitated by adding anhydrous ethanol and then harvested by centrifugation. To synthesize the biocompatible nanozyme, CeMn NPs were coated with DPSE-mPEG^2k^ copolymer. Briefly, mPEG^2k^-DPSE (50 mg, 1.85 × 10^−2^ mM) dissolved in 2.0 ml of chloroform was mixed with 10 mg of CeMn NP in chloroform. After stirring for 2 h, the solvents were evaporated by a rotary evaporator and then 10.0 ml of ddH_2_O was added and dispersed by probe sonication to obtain the water-soluble nanozyme CeMn-PEG. The precipitate was removed by filtration, and excess mPEG^2k^-DPSE was removed by dialysis against a 7,000-Da molecular weight cutoff bag filter.

### Characterization

Transmission electron microscope (TEM) images and selected-area electron diffraction (SAED) pattern were conducted using a TECNAI G2 F20 S-TWIN (FEI, Hillsboro, Oregon, USA) TEM operated at 200 kV. The elemental compositional analysis of CeMn NP was carried out using an EDS system attached to TEM. X-ray powder diffraction (XRD) patterns were recorded on a SmartLab XRD instrument (Rigaku Corporation, Tokyo, Japan). 2θ range was from 5° to 90° with Cu Kα radiation (λ = 0.154 nm), operated at 30 mA and 40 kV. Phase identification was performed by MDI JADE 5.0 software. The x-ray photoelectron spectra (XPS) were recorded using an ESCALAB 250Xi spectrometer (Thermo Fisher Scientific, MA, USA) equipped with monochromatized Al Kα radiation (hv = 1,468.6 eV). All of the binding energies were referenced to the C1s peak at 284.8 eV. The hydrodynamic diameters and zeta potentials of CeMn-PEG were measured by DLS (Zetasizer Pro, Malvern, UK) performed at 25 °C. The concentration of Ce and Mn was determined by inductively coupled plasma–mass spectrometry (ICP-MS; iCAP TQ, Thermo Fisher Scientific, Bremen, Germany) after digestion using HNO_3_ (60%) and H_2_O_2_ (30%).

### Free radical scavenging and enzyme-mimicking activity assay of CeMn-PEG

The dilution of the CeMn-PEG nanoparticle samples for the designed experiments is consistently performed using Milli-Q water.

$$\text{Scavenging rate}\;(\%)=\left(1-A_{s}/A_{0}\right)\times 100$$ (1)

where *A*_s_ is the sample absorbance and *A*_0_ is a sample concentration of 0.

### DPPH radical scavenging activity assay of CeMn-PEG

To assess radical scavenging activity of CeMn-PEG, 0.2 ml of DPPH solution was mixed with 0.2 ml of test sample in stoppered tube for half an hour. Then, the scavenging efficiency of CeMn-PEG against DPPH free radicals was determined by using a spectrophotometer at a wavelength of 517 nm.

### ABTS·^+^ radical scavenging assay of CeMn-PEG

The ABTS radical scavenging assay was conducted according to the protocol from the manufacturer. Briefly, ABTS was dissolved in ddH_2_O to prepare a 7 mM stock solution. ABTS solution was mixed with 12.25 mM potassium persulfate (K_2_S_2_O_8_) solution with a 5:1 (v/v) ratio and allowed to stand at room temperature in the dark for 12 h to prepare ABTS radical cation (ABTS**·**^+^). For the assay, the ABTS**·**^+^ solution was diluted with anhydrous ethanol to an absorbance between 0.8 and 0.9 at 734 nm and was stable at room temperature. Then, 100 μl of the diluted ABTS**·**^+^ solution and 5 μl of the test sample were mixed in 96-well plate and allowed to stand in the dark for 7 min. Then, the residual amount of ABTS**·**^+^ was measured by using a spectrophotometer at a wavelength of 734 nm.

### Hydroxyl radical scavenging experiment of CeMn-PEG

Following the manufacturer’s protocol of Hydroxyl Radical Detection Assay Kit, CeMn-PEG samples were mixed with the assay reagents and incubated at 37 °C for 10 min. Then, the hydroxyl radical scavenging rate of CeMn-PEG was calculated according to the absorbance measured at a wavelength of 904 nm by spectrophotometer.

### Superoxide anion scavenging experiment of CeMn-PEG

The superoxide dismutase (SOD) enzyme activity (O_2_**·**^−^) of CeMn-PEG was assessed by using Beyotime’s Total Superoxide Dismutase Assay Kit with WST-8 (Beyotime, Shanghai, China) following the manufacturers protocol. Then, the SOD scavenging rate of CeMn-PEG was calculated according to the absorbance measured by spectrophotometer.

### CAT-like activity of CeMn-PEG

After 500 μM H_2_O_2_ solution was mixed with CeMn-PEG, this mixed solution was stirred at 50 rpm. Then, the dissolved oxygen curve of this mixed solution was determined by using a dissolved oxygen meter (JBPJ-608, Shanghai, China).

### Mouse model of IDD

A total of 72 C57/BL6 mice (male, 7 to 8 weeks) were randomly divided into 3 groups: sham group (non-needle puncture, without any injection), puncture group (puncture with injection of PBS), and CeMn-PEG treatment group (puncture with injection of CeMn-PEG). After the mouse was anesthetized by using isoflurane inhalation, we shaved and cleaned the skin of abdomen. Then, the spine was exposed through a midline ventral longitudinal incision and the disc (L5–L6) was accurately localized based on vascular anatomy and punctured by a 33-gauge needle [82,83]. At the time of puncturing, the puncture group and CeMn-PEG treatment group were injected with 5 μl of PBS and CeMn-PEG (1 μg/ml), respectively. After the surgery was completed, the incision was sutured and disinfected with iodine volts. Then, the mouse was placed on heat plate before waking up from anesthesia. Finally, all mice were allowed to feed, move, and sleep freely.

### MRI and x-ray assessment

MRI scanning and x-rays were performed at 3 and 6 weeks after surgery. Eight mice from each group were anesthetized as described above and then were placed in a 9.4-T MRI scanner (9.4T; BRUKER BioSpec 94/20, Germany) to obtain T2-weighted images and in a small-animal digital x-ray scanner (Medsinglong, China) to obtain x-ray images. The T2-weighted signal parameter was set as in a previous study [82]: echo time, 75 ms; spin echo repetition time, 4,000 ms. The Pfirrmann MRI grading score was assessed by a blinded spine surgeon according to the Pfirrmann classification (1 point = grade I; 2 points = grade II; 3 points = grade III; 4 points = grade IV; and 5 points = grade V). As for x-ray assessment, imaging software (ImageJ, NIH) was used for measuring the height of each disc and the adjacent vertebrae and the disc height index (DHI) was calculated as in a previous method [84].

### Histopathologic analysis

At 3 and 6 weeks after the operation, mice of each group (*n* = 8) were euthanized via pentobarbital sodium anesthesia and were sacrificed to obtain L5–L6 discs with the adjacent vertebrae. After fixing in 4% paraformaldehyde (PFA) for 48 h at 4 °C and decalcification in 10% EDTA in PBS at 4 °C for 2 weeks, all the specimens were embedded in paraffin and sectioned to a 5-mm thickness along the midsagittal plane. The slides of each disc were stained with hematoxylin and eosin (H&E) and Safranin O/Fast Green staining following the standard protocols. Images were captured by using an optical microscope and scaled according to the previous criteria [85].

### Von Frey test

Hindpaw mechanical sensitivity was employed as an index to evaluate the radiating pain elicited by needle puncture, quantified through the application of von Frey filaments (model Bio-VF-M, manufactured by Bioseb). Briefly, mice were placed in an opaque plastic cube on a wire mesh platform for 1.5 h prior to testing and 50% paw withdrawal threshold of the left paw was calculated according to the up-down method as previously reported [86].

### Cell culture

Primary NP and RAW264.7 cell lines were purchased from Ke Lei Biotechnology Co. Ltd. (#CR0056 and #C0158, Shanghai, China). These cells were cultured in Dulbecco’s modified Eagle’s medium (DMEM) supplemented with 10% fetal bovine serum (FBS) and 1% penicillin–streptomycin.

### Cell viability analysis

Enhanced Cell Counting Kit 8 (WST-8/CCK8; Elabscience, Wuhan, China) was used to assess the cytotoxic effects of CeMn-PEG in vitro following the manufacturer’s protocol. NP and RAW264.7 cells were seeded on a 96-well plate at a density of 1 × 10^5^ cells per well, followed by a 24-h incubation. Subsequently, various concentrations of CeMn-PEG (0, 0.25, 0.5, 1, and 2 μg/ml) were added into the culture medium and incubated for an additional 48 h. Later, all the wells were washed with fresh PBS 3 times. Later, after 100 μl of DMEM containing 10 μl of CCK8 solution in each well was incubated at 37 °C for 2 h, the absorbance was measured at a wavelength of 450 nm by using Multiskan FC (Thermo Fisher Scientific).

### Cell treatment

NP cells (1 × 10^6^/well) were seeded on a 6-well plate and incubated at 37 °C for 24 h. After NP cells were pretreated with CeMn-PEG (1 μg/ml) or PBS for 6 h, NP cells then were treated with 200 μM H_2_O_2_ for 6 h. Following this stimulation of H_2_O_2_, NP cells were collected for evaluating the protective effect of CeMn-PEG on NP cells by using qRT-PCR, cellular immunofluorescence staining, flow cytometry, and Western blot analysis.

### Establishment of NP cells/RAW264.7 cells cocultured system

The transwell chambers (Corning Falcon) were used for establishing the coculture system of NP and RAW264.7 cells as shown in Fig. 5A to evaluate the effect of CeMn-PEG on the interactions between RAW264.7 and NP cells. Briefly, RAW264.7 cells (2.5 × 10^5^/well) were seeded into the upper chambers and NP cells (2.5 × 10^5^/well) were seeded into the bottom chambers. Following coculturing at 37 °C for 24 h, RAW264.7 cells were pretreated with CeMn-PEG (1 μg/ml) or PBS for 6 h. Then, both NP and RAW264.7 cells were stimulated with 200 μM H_2_O_2_ or PBS for 6 h. Subsequently, NP and RAW264.7 cells were collected for assessing the macrophage polarization and protective effect of RAW264.7 cells on NP cells by using qRT-PCR, cellular immunofluorescence staining, and flow cytometry. Meanwhile, a negative group was established in which the cocultured RAW264.7 cells were replaced by the treatment of PBS.

### Determination of the ROS scavenging capacity of CeMn-PEG in vitro

Dihydroethidium (Beyotime, Shanghai, China) was used to detect the level of intracellular ROS in vitro following the manufacturer’s protocol. After NP and RAW264.7 cells were seeded on slides and were treated as previously described, 1 ml of DHE (1:1,000) was added to incubate cells in 12-well plates at 37 °C for 20 min. The levels of ROS were reflected by a fluorescence microscope (ECLIPSE Ts2, Nikon) and analyzed by using ImageJ.

### Administration of the autophagy inducer rapamycin

The autophagy inducer rapamycin (MCE, #AY-22989) was employed for determining whether the protective effect of CeMn-PEG on H_2_O_2_-induced apoptosis of NP cells could be inhibited by inducing autophagy. After NP cells (1 × 10^6^/well) were seeded on a 6-well plate and incubated at 37 °C for 24 h, NP cells were pretreated with rapamycin (50 nM) or PBS for 6 h before the treatment with CeMn-PEG (1 μg/ml) or PBS for 6 h. Subsequently, NP cells were stimulated with 200 μM H_2_O_2_ for 6 h. Then, NP cells were collected for assessing apoptosis rate and MMP.

### Quantitative RT-PCR

qRT-PCR was used to quantify the mRNA expression of collagen II, aggrecan, MMP13, ADAMTS5, IL-1β, IL-4, IL-6, and IL-10 according to the manufacturer’s protocol. Briefly, after total RNA of treated NP and RAW264.7 cells was extracted by using TRIzol reagent (Invitrogen), the concentration of RNA was determined and the cDNA was synthesized by using reverse transcriptase. Then, a 10-μl reaction system containing 5-μl SYBR Master mix (Vazyme), 0.25-μl primer, and 4.5-μl diluted cDNA was applied for PCR amplification. Finally, the relative gene expressions were analyzed using the comparative CT (cycle threshold) method. The primers used are listed in Table Eq. 1.

### Western blot analysis

Western blotting was performed according to previous protocols. The total protein of treated NP and RAW264.7 cells was extracted by using radioimmunoprecipitation assay (RIPA) buffer containing 1 mM phenylmethanesulfonyl fluoride (PMSF), and then the BCA protein assay kit (Beyotime) was used to determine the concentration of extracted protein. Nuclear and cytoplasmic proteins were extracted by using the Nuclear and Cytoplasmic Protein Extraction Kit according to the manufacturer’s protocol. Then, 20-μg protein of each sample was separated by using sodium dodecyl sulfate–polyacrylamide gel electrophoresis and transferred to a nitrocellulose membrane (Life Technologies, Gaithersburg, MD, USA). Then, the membranes were blocked in tris-buffered saline with Tween 20 (TBST) solution with 5% bovine serum albumin for 1 h before incubation with primary antibodies overnight at 4 °C. The primary antibodies were as follows: β-actin (1:2,000, Proteintech), type II collagen (1:1,000, BIOSS), aggrecan (1:1,000, Proteintech), MMP13 (1:1,000, Affinity), IL-4 (1:1,000, Affinity), ADAMTS5 (1:1,000, Affinity), IL-1β (1:1,000, Affinity), IL-6 (1:2,000, Affinity), and IL-10 (1:1,000, Affinity). Following the incubation with respective secondary antibodies for 2 h at room temperature, AlphaEaseFC Software (Alpha Innotech, USA) was used to detect and analyze the signals. β-Actin was used for normalization.

### Cellular immunofluorescence staining

After NP and RAW264.7 cells were plated on slides and were treated as previously described, samples were fixed with 4% PFA for 15 min at room temperature and then washed with fresh PBS for 5 min 3 times. Then, samples were treated with PBS containing 0.25% Triton X-100 for 10 min and blocked with 10% FBS. Subsequently, samples were incubated with primary antibodies against CD86 (Bioss, #bs-1035R, 1:300), CD206 (Affinity, #DF4149, 1:300), C-caspase3 (Affinity, #AF7022, 1:300), and LC3 (CST, #83506S, 1:300) overnight at 4 °C. The next day, samples were washed with fresh PBS 3 times for 5 min and then incubated with Cy3-conjugated anti-immunoglobulin G (IgG) (BOSTER, #BA1031, 1:500) or FITC-conjugated anti-IgG (BOSTER, #BA1105, 1:300) secondary antibodies for 1 h. Subsequently, the samples were stained with 4′,6-diamidino-2-phenylindole (DAPI) (Invitrogen) and observed by using a fluorescence microscope (ECLIPSE Ts2, Nikon).

### Immunofluorescence staining in DRG

At 3 and 6 weeks after the operation, mice of each group (*n* = 4) were euthanized via pentobarbital sodium anesthesia and sacrificed to obtain bilateral L4–L6 DRGs. After fixing with 4% PFA overnight at 4 °C, DRGs were dehydrated in 30% sucrose solution overnight at 4 °C and embedded in optimal cutting temperature compound. Samples were then sectioned to a 12-mm thickness with microtome. The immunofluorescence staining process was similar to cellular immunofluorescence staining. Primary antibodies against CGRP (Abcam, #ab189786, 1:500) and Cy3-conjugated anti-IgG (BOSTER, #BA1031, 1:500) secondary antibodies were used in the following staining. For each DRG section (from 4 mice), the number of CGRP-positive neurons and total neurons was quantified by using ImageJ as in a previous study [87]. The percentage of CGRP-IR DRG neurons was the number of CGRP-positive neurons divided by the total number of DRG neurons.

### Flow cytometry

After NP and RAW264.7 cells were treated as previously described, the digested single-cell suspension was incubated with flow cytometry antibodies, CD86-FITC (fluorescein isothiocyanate), and CD206-APC (allophycocyanin) at room temperature in the dark for 1 h. The V-APC/7-AAD Apoptosis Detection Kit (KeyGEN) was used according to the manufacturer’s instruction. Flow cytometry (Beckman Coulter) was then used to evaluate the level of CD86, CD206, and cell apoptosis.

### MMP assessment

MMP was determined by using mitochondrial membrane potential assay kit with JC-1 (JC-1 Assay Kit, C2006, Beyotime) following the manufacturer’s instructions. Briefly, after the incubation with JC-1 working solution at 37 °C for 20 min, the NP cells with different treatment were centrifuged at 300*g* at 4 °C for 4 min and then the culture supernatant was discarded. After washing twice and resuspending with JC-1 buffer solution, the NP cells were collected for assessing the MMP using flow cytometry.

### Statistical analysis

GraphPad Prism 8.0 was used for statistics. The data were expressed as mean ± standard error of measurement (SEM). One-way or 2-way analysis of variance (ANOVA) with Tukey’s multiple comparison test was used for analyzing normally distributed and equal variance data of multiple groups. Kruskal–Wallis test with Dunn’s multiple comparisons was used for analyzing ranked data of multiple groups. *P* < 0.05 was considered to indicate statistically significant differences. Figures were created with BioRender.com.

## Data Availability

No datasets were generated or analyzed during the current study.
