# Supplementary material for: Custom-Made Ce–Mn Bimetallic Nanozyme for the Treatment of Intervertebral Disc Degeneration by Inhibiting Oxidative Stress and Modulating Macrophage M1/M2 Polarization
Source: Biomater Res. 2024 Dec 23;28:0118. doi: 10.34133/bmr.0118 (PMC11665849; doi:10.34133/bmr.0118)
Supplement: Supplementary 1 — Figs. S1 to S4 Table S1 [file bmr.0118.f1.docx]

Supporting Information

Custom-made Ce-Mn bimetallic nanozyme for the treatment of IDD via inhibiting oxidative stress and modulating macrophage M1/M2 polarization

Jianwei Wu^1^, Zhenhao Chen^1^, Han Huang^1^, Hongwei Wang^1^, Xianghe Wang^1^, Zian Lu^1^, Haocheng Xu^1^, Xiaosheng Ma^*1^, Feng Zeng^*2^, Hongli Wang^*1^


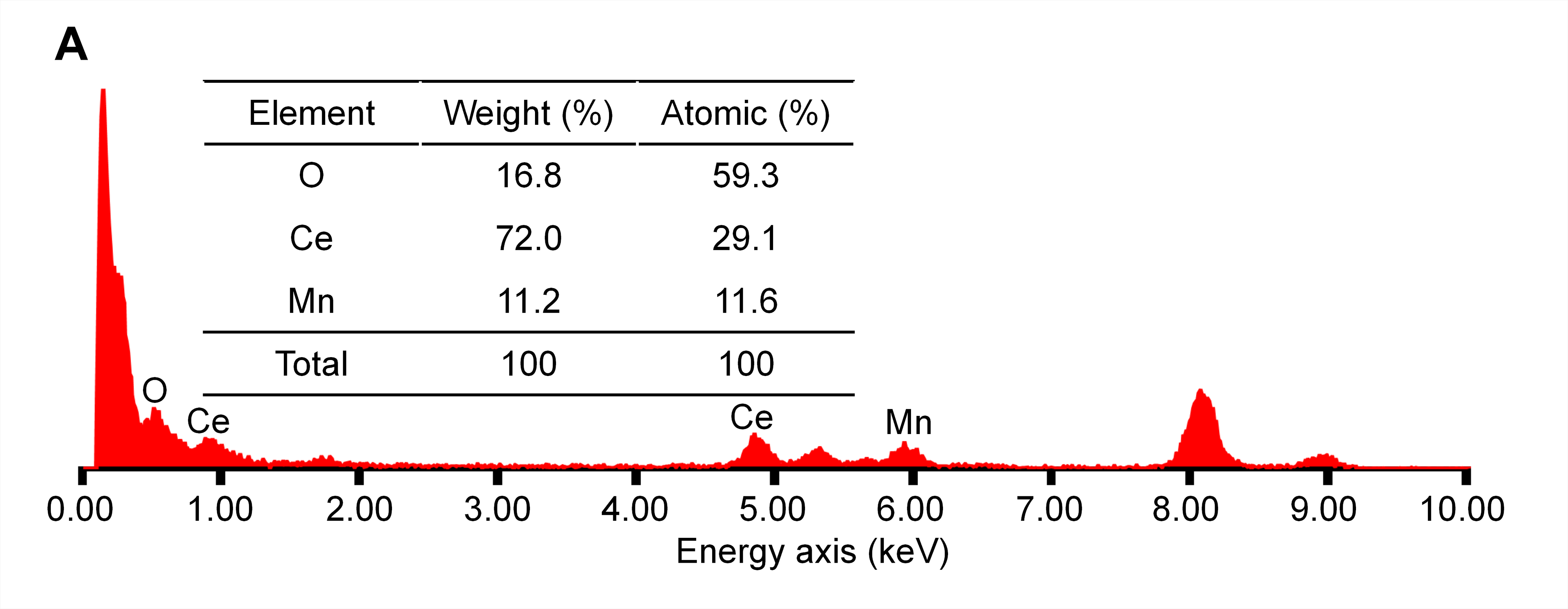


Figure S1. (A) Chemical composition of the CeMn-PEG measured by energy dispersive spectrometer (EDS) analysis. Inset table: the weight percentages and atomic percentages of the Ce and Mn, respectively.


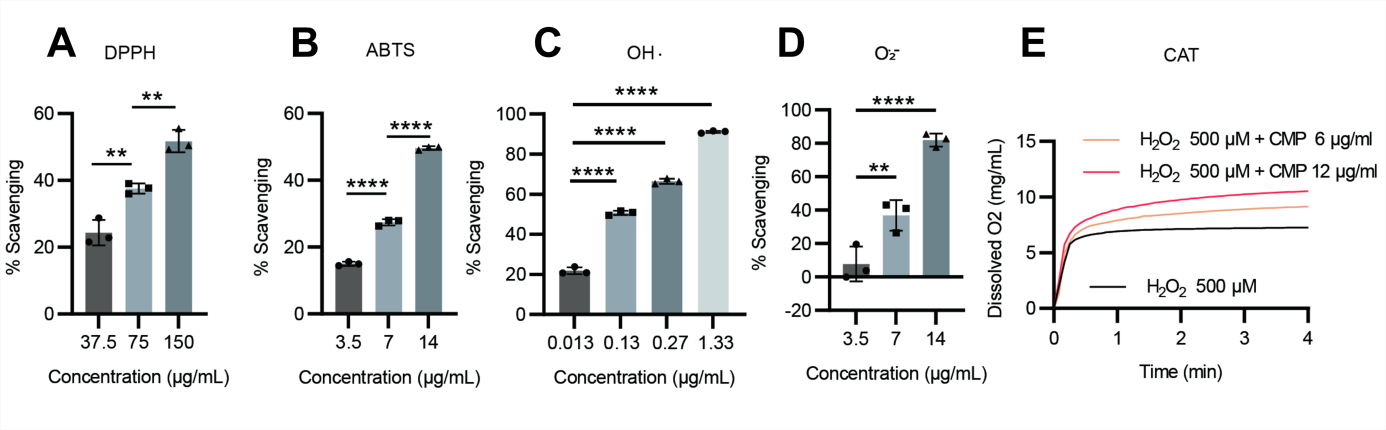


Figure S2. Enzyme-like activity and antioxidant capacity of CeMn-PEG. (A) DPPH-free radical scavenging activity; (B) ABTS^·+^ radical scavenging activity; (C) Hydroxyl radical scavenging activity; (D)Superoxide anion scavenging activity; (E) Catalase-like activity, CMP: CeMn-PEG. Values are presented as a percentage of the control. Data are presented as the mean ± SEM. *P < 0.05, **P < 0.01, ***P < 0.001, n = 3.


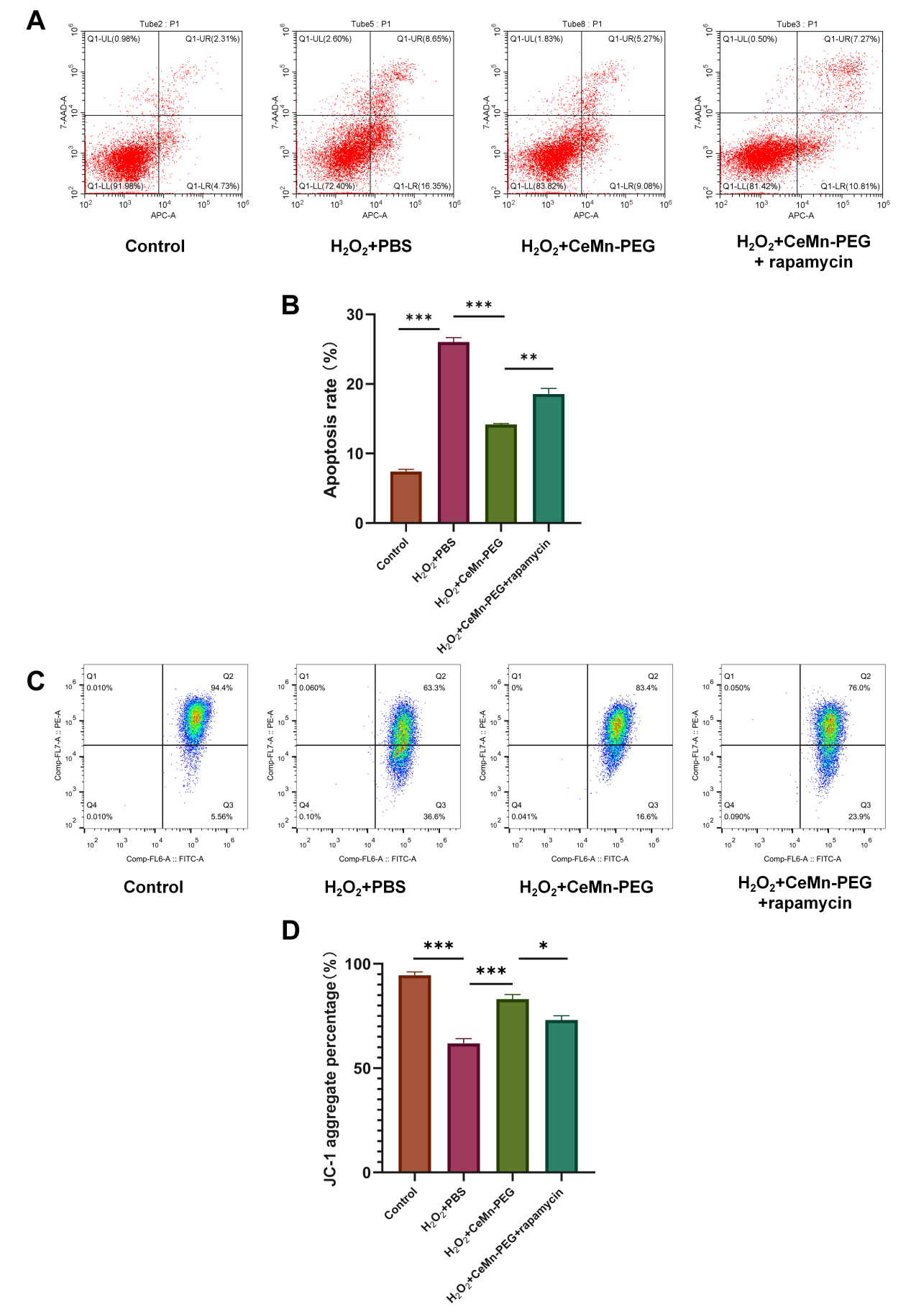


Figure S3. Apoptosis rate and MMP of NP cells in each group estimated by flow cytometry. (A, B) Apoptosis rate of NP cells in each group NP estimated by flow cytometry analysis. (C, D) The MMP in NP cells estimated by flow cytometry using JC-1 staining. Data are presented as the mean ± SEM. *P < 0.05, **P < 0.01, ***P < 0.001, n = 3.


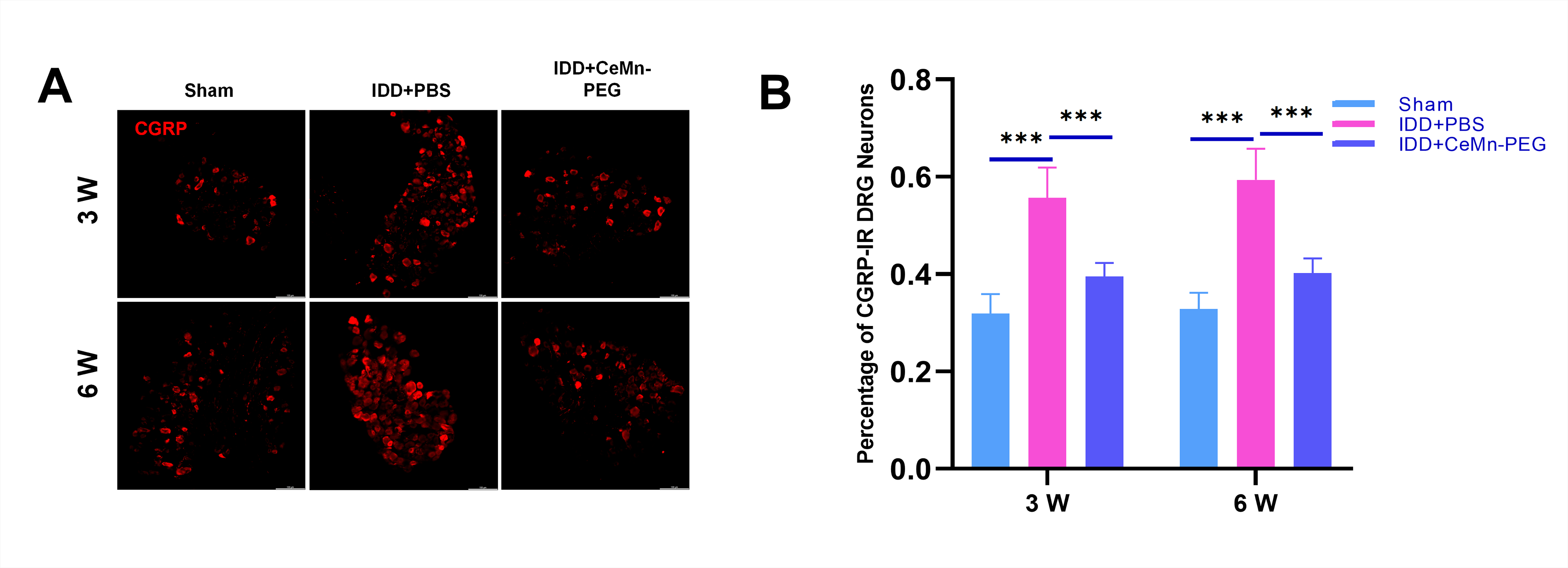


Figure S4. (A) Representative immunofluorescence images of CGRP (red). (B) The percentage of CGRP-IR DRG neurons in each group estimated by immunofluorescence staining. Scale bar, 100 μm. Data are presented as the mean ± SEM. *P < 0.05, **P < 0.01, ***P < 0.001, n = 4.

Table S1. Primer sequences used in qRT-PCR

| **Gene** | **Primer** | **Sequence (5'-3')** | **PCR Products** |
| --- | --- | --- | --- |
| β-actin | Forward | CACGATGGAGGGGCCGGACTCATC | 240 bp |
|  | Reverse | TAAAGACCTCTATGCCAACACAGT |  |
| Collagen II | Forward | TGACTTTCCTCCGTCTACTGTC | 249 bp |
|  | Reverse | AGGTCTTCTGTGATCGGTACTC |  |
| Aggrecan | Forward | CCTTCGCTCCAATGACTC | 101 bp |
|  | Reverse | GGAACACAATGCCTTTTACT |  |
| MMP13 | Forward | CCCGAGACCTCATGTTCATCT | 243 bp |
|  | Reverse | CTTCTTCTATGAGGCGGGGAT |  |
| Adamts5 | Forward | CAACTCCGTGTGTGTCCG | 267 bp |
|  | Reverse | TCGCCAGTTTTCTTCTTTAGG |  |
| IL-1β | Forward | CCTGTGTGATGAAAGACGGC | 218 bp |
|  | Reverse | TATGTCCCGACCATTGCTGT |  |
| IL-6 | Forward | GGAGTTCCGTTTCTACCTGGA | 209 bp |
|  | Reverse | TGGTCCTTAGCCACTCCTTC |  |
| IL-4 | Forward | CCAGGTCACAGAAAAAGGG | 149 bp |
|  | Reverse | AGCACGGAGGTACATCACG |  |
| IL-10 | Forward | CCTGGCTCAGCACTGCTATG | 101 bp |
|  | Reverse | ACTGGGAAGTGGGTGCAGTT |  |
